# O-GlcNAcylation stimulates the deubiquitination activity of USP16 and regulates cell cycle progression

**DOI:** 10.1016/j.jbc.2024.107150

**Published:** 2024-03-09

**Authors:** Jianxin Zhao, Jie Hua, Yahui Zhan, Chunxu Chen, Yue Liu, Liqian Yang, Haiying Wang, Hengbin Wang, Jing Li

**Affiliations:** 1 Beijing Key Laboratory of DNA Damage Response and College of Life Science, Capital Normal University, Beijing, China; 2 Department of Biochemistry and Molecular Genetics, University of Alabama at Birmingham, Birmingham, Alabama, USA; 3 School of Life Sciences, Fudan University, Shanghai, China; 4 Massey Cancer Center, Virginia Commonwealth University, Richmond, Virginia, USA; 5 Department of Bioengineering, Virginia Commonwealth University, Richmond, Virginia, USA; 6 Beijing Key Laboratory of Protein Posttranslational Modifications and Cell Function, Department of Biochemistry and Biophysics, School of Basic Medical Sciences, Peking University Health Science Center, Beijing, China; 7 Division of Hematology, Oncology, and Palliative Care, Department of Internal Medicine, Virginia Commonwealth University, Richmond, Virginia, USA

**Keywords:** mitosis, histone H2A ubiquitination, O-GlcNAcylation, cytokinesis, PLK1

## Abstract

Histone 2A monoubiquitination (uH2A) underscores a key epigenetic regulation of gene expression. In this report, we show that the deubiquitinase for uH2A, ubiquitin-specific peptidase 16 (USP16), is modified by O-linked N-acetylglucosamine (O-GlcNAc). O-GlcNAcylation involves the installation of the O-GlcNAc moiety to Ser/Thr residues. It crosstalks with Ser/Thr phosphorylation, affects protein–protein interaction, alters enzyme activity or protein folding, and changes protein subcellular localization. In our study, we first confirmed that USP16 is glycosylated on Thr203 and Ser214, as reported in a previous chemoenzymatic screen. We then discovered that mutation of the O-GlcNAcylation site Thr203, which is adjacent to deubiquitination-required Cys204, reduces the deubiquitination activity toward H2AK119ub *in vitro* and in cells, while mutation on Ser214 had the opposite effects. Using USP16 Ser552 phosphorylation-specific antibodies, we demonstrated that O-GlcNAcylation antagonizes cyclin-dependent kinase 1–mediated phosphorylation and promotes USP16 nuclear export. O-GlcNAcylation of USP16 is also required for deubiquitination of Polo-like kinase 1, a mitotic master kinase, and the subsequent chromosome segregation and cytokinesis. In summary, our study revealed that O-GlcNAcylation of USP16 at Thr203 and Ser214 coordinates deubiquitination of uH2A and Polo-like kinase 1, thus ensuring proper cell cycle progression.

The addition of N-acetylglucosamine (GlcNAc) onto Ser/Thr residues of nuclear or cytosolic proteins is catalyzed by the sole O-linked N-acetylglucosamine (O-GlcNAc) transferase (OGT) (1, 2). The precise function of O-GlcNAcylation has been a captivating question for both biologists and chemists (1, 2).

O-GlcNAc crosstalks with other post-translational modifications, *e.g.*, phosphorylation (2) and ubiquitination (3). It regulates the nuclear translocation of proteins across the nuclear pore complex (4). OGT has also been studied in the field of epigenetics (5, 6, 7), where it modifies histones *in vitro* and *in vivo* (8). For instance, O-GlcNAcylated histone H2B Ser112 facilitates its monoubiquitination (9), and this glycosylation event is enhanced by TET2-OGT binding (10). Additionally, H3pS10, a mitotic marker, has been proposed to be under the stringent control of O-GlcNAc, as H3 Ser10 is O-GlcNAcylated (11). Recently, H4S47 O-GlcNAcylation is demonstrated to be important for replication origin activation (12). Apart from histones, OGT substrates also include “writers” and “erasers” of the histone code. Mixed lineage leukemia 5, the histone H3K4 methyltransferase, is O-GlcNAcylated (13). Also, the enhancer of zeste homolog 2 protein is O-GlcNAcylated (14, 15), which stabilizes H3K27me3. Therefore, O-GlcNAc modification plays an integral role in the histone code.

Recent chemoproteomic O-GlcNAc studies have revealed many new OGT substrates (16), functions of which are yet to be investigated. Specifically, a combination of biochemical fractionation and click chemistry has given rise to many interesting results (17). One substrate that caught our attention was ubiquitin-specific peptidase 16 (USP16, also named as UBP-M). As a deubiquitinase (DUB), USP16 catalyzes the deubiquitination of histone 2A monoubiquitination (uH2A) at K119 (18). It is crucial for cell cycle progression and is pivotal for hematopoietic stem cell differentiation (19, 20). During animal development, uH2A is instrumental for *Hox* gene silencing and X chromosome inactivation. During oocyte maturation, USP16 is essential for zygotic genome activation (21). In peripheral T cells, USP16 is instrumental for cell maintenance and proliferation by deubiquitinating calcineurin A (22). On the therapeutic side, USP16 is oncogenic in the K-RAS-driven lung cancer *via* p38 and JAK1 (23) and in castration-resistant prostate cancer cell *via* c-Myc (24). In glioblastoma, the lncRNA (lncEPAT) inhibits the chromatin recruitment of USP16 and represses target gene expression (25). USP16 is also identified recently to be a potential target in an Alzheimer’s model (26).

During mitosis, cyclin-dependent kinase 1 (Cdk1) phosphorylates USP16 at Ser552 and regulates its nuclear import by the exportin chromosome region maintenance 1 (Crm1) (27, 28). USP16 prefers uH2A substrates on nucleosomes *in vitro*. The USP16-mediated uH2A deubiquitination is a prerequisite for H3pS10, and thus mitotic progression (18). Another mitotic substrate of USP16 is Polo-like kinase 1 (PLK1) (28), which is a master kinase orchestrating many mitotic events, including mitotic entry, spindle assembly, chromosome segregation, and cytokinesis (29). USP16 deubiquitinates PLK1, increasing PLK1-kinetochore association to ensure proper chromosome alignment during early mitosis (28).

Herein, we show that USP16 is O-GlcNAcylated at two sites, Thr203 and Ser214. Using phospho-specific antibodies, we demonstrate that USP16 O-GlcNAcylation antagonizes CDK1-mediated phosphorylation and thus nuclear shuttling. *Via in vitro* and in cell histone deubiquitination assays, we found that the two O-GlcNAcylation sites have opposing effects in promoting the DUB activity of USP16. Moreover, when cells were synchronized in early mitosis, the O-GlcNAc-deficient T203A/S214A (2A) mutant attenuates its DUB activity against PLK1, leading to misaligned chromosomes and cytokinesis defects. Our work not only identifies a new OGT substrate involved in cell cycle control and epigenetics, but also highlights the potential of using a combination of biochemistry and chemical biology approaches to identify more O-GlcNAc targets.

## Results

### USP16 is O-GlcNAcylated at Thr203 and Ser214

**Figure 1:**
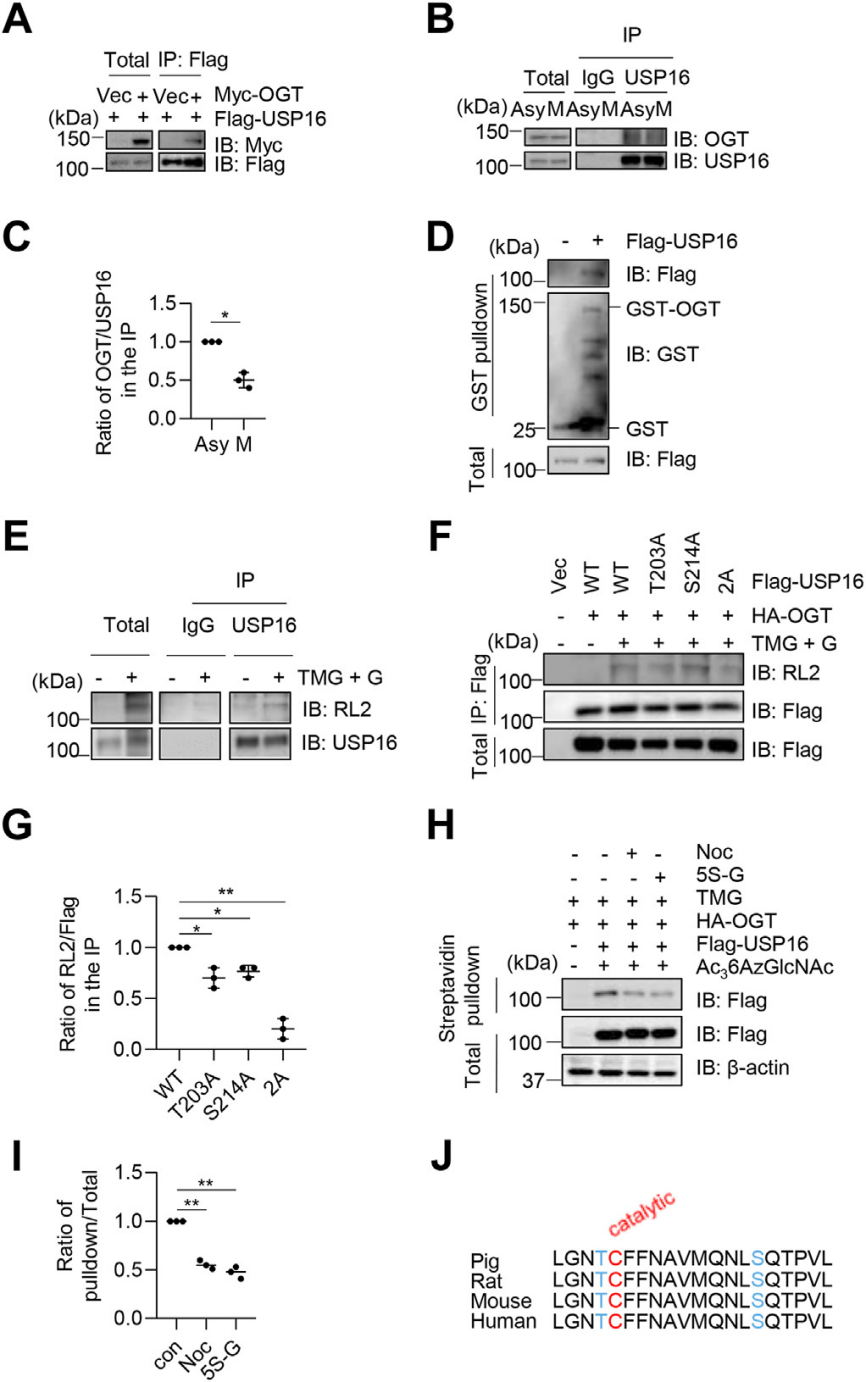
**USP16 is O-GlcNAcylated at Thr-203 and Ser-214.***A*, HeLa cells were transfected with Myc-OGT and Flag-USP16 plasmids. The cell lysates were immunoprecipitated with anti-Flag antibodies and immunoprecipitated with anti-Myc antibodies. *B*, HeLa cells were synchronized to M phase by nocodazole (Noc) or untreated. The cellular lysates were subject to immunoprecipitation with anti-USP16 antibodies and then immunoblotted with anti-OGT antibodies. *C*, quantitation of (*B*). A *t* test was used in the statistics. *D*, cells were transfected with Flag-USP16 plasmids or vectors. Then recombinant GST-OGT proteins were incubated with the cellular lysates. *E*, cells were enriched for O-GlcNAc proteins by treating with TMG plus glucose (TMG + G) as previously described (32). The cell lysates were immunoprecipitated with anti-USP16 antibodies and immunoblotted with RL2. *F*, cells were transfected with vector, Flag-USP16-WT, T203A, S214A, and T203AS214A (2A) plasmids, together with HA-OGT, then treated with TMG + G. *G*, quantitation of (*F*). *H*, cells were transfected with Flag-USP16 and HA-OGT, then treated with 200 μmol/l Ac_3_6AzGlcNAc and 5 μmol/l TMG, treated or not treated with Noc or 5S-G (OGT inhibitor) as previously described (40). *I*, quantitation of (H). A one-way ANOVA was used in the statistics. *J*, the potential O-GlcNAc sites flank the catalytic Cys-204 site and are conserved. ∗indicates *p* < 0.05; ∗∗indicates *p* < 0.01. 5S-G, acetyl-5S-GlcNAc; Asy, asynchronous; M, mitosis; OGT, O-GlcNAc transferase; TMG, Thiamet-G; USP16, ubiquitin-specific peptidase 16.

A recent investigation using azide-alkyne click chemistry combined with extensive biochemical fractionation revealed that USP16 is O-GlcNAcylated at Thr203 and Ser214 (17). To confirm this result, we first investigated whether USP16 interacts with OGT, the enzyme responsible for O-GlcNAcylation. For this purpose, we cotransfected HeLa cells with Myc-OGT and Flag-USP16 and performed co-immunoprecipitation (co-IP) assays with the anti-Flag antibody (Fig. 1*A*). USP16 was found to co-IP with OGT (Fig. 1*A*). To investigate whether USP16 interacts with OGT endogenously, we performed experiments with the anti-USP16 antibody. USP16 was found to co-IP with OGT (Fig. 1, *B* and *C*). Interestingly, we discovered that the interaction between OGT and USP16 was reduced in lysates prepared from the mitotic (M) phase of cells (Fig. 1*C*), raising a possibility that the interaction of USP16 and OGT may be cell-cycle dependent. The interaction was also confirmed by revealing that GST-OGT, but not GST itself, pulled down Flag-USP16 expressed in HeLa cells (Fig. 1*D*).

We then investigated whether USP16 is O-GlcNAcylated. We treated cells with the OGA inhibitor, Thiamet-G (TMG) and glucose, to increase the levels of protein O-GlcNAcylation (30, 31) (abbreviated at TMG + G). We also included TMG during cell lysis and immunoprecipitation (IP) procedures. Under this condition, the immunoprecipitated USP16 showed an apparent O-GlcNAc band (Fig. 1*E*). USP16 O-GlcNAcylation occurs on both Thr203 and Ser214, as mutations of both T203A and S214A simultaneously abolished the O-GlcNAcylation band while mutating either one had little effects (Fig. 1, *F* and *G*). Click chemistry was also utilized to examine USP16 O-GlcNAcylation (Fig. 1, *H* and *I*). We observed that nocodazole and acetyl-5S-GlcNAc (5S-G) (OGT inhibitor) treatment dampened the O-GlcNAcylation levels (Fig. 1, *H* and *I*). Notably, Cys204 is the residue required for USP16 deubiquitination activity, with Thr203 adjacent to it and Ser214 close by, raising the possibility that O-GlcNAcylation may regulate USP16’s deubiquitination activity (Fig. 1*J*).

### O-GlcNAcylation antagonizes CDK1-mediated USP16 Ser552 phosphorylation

Our previous study found that CDK1-mediated phosphorylation of USP16 at Ser552 occurs as cells enter the G_2_/M phase, which facilitates its nuclear localization and helps to remove uH2A for chromosome condensation when cells progress into the M phase (27, 28). This phosphorylation may also prime USP16 for further phosphorylation by PLK1 at Ser330 and Ser386 (28). Previous reports from our lab suggest that CDK1-mediated phosphorylation is counteracted by O-GlcNAcylation of CDC20 homolog 1 (Cdh1) (31) and myosin phosphatase targeting subunit 1 (MYPT1) (32). Therefore, we investigated whether O-GlcNAcylation antagonizes CDK1-mediated USP16 Ser552 phosphorylation.

**Figure 2:**
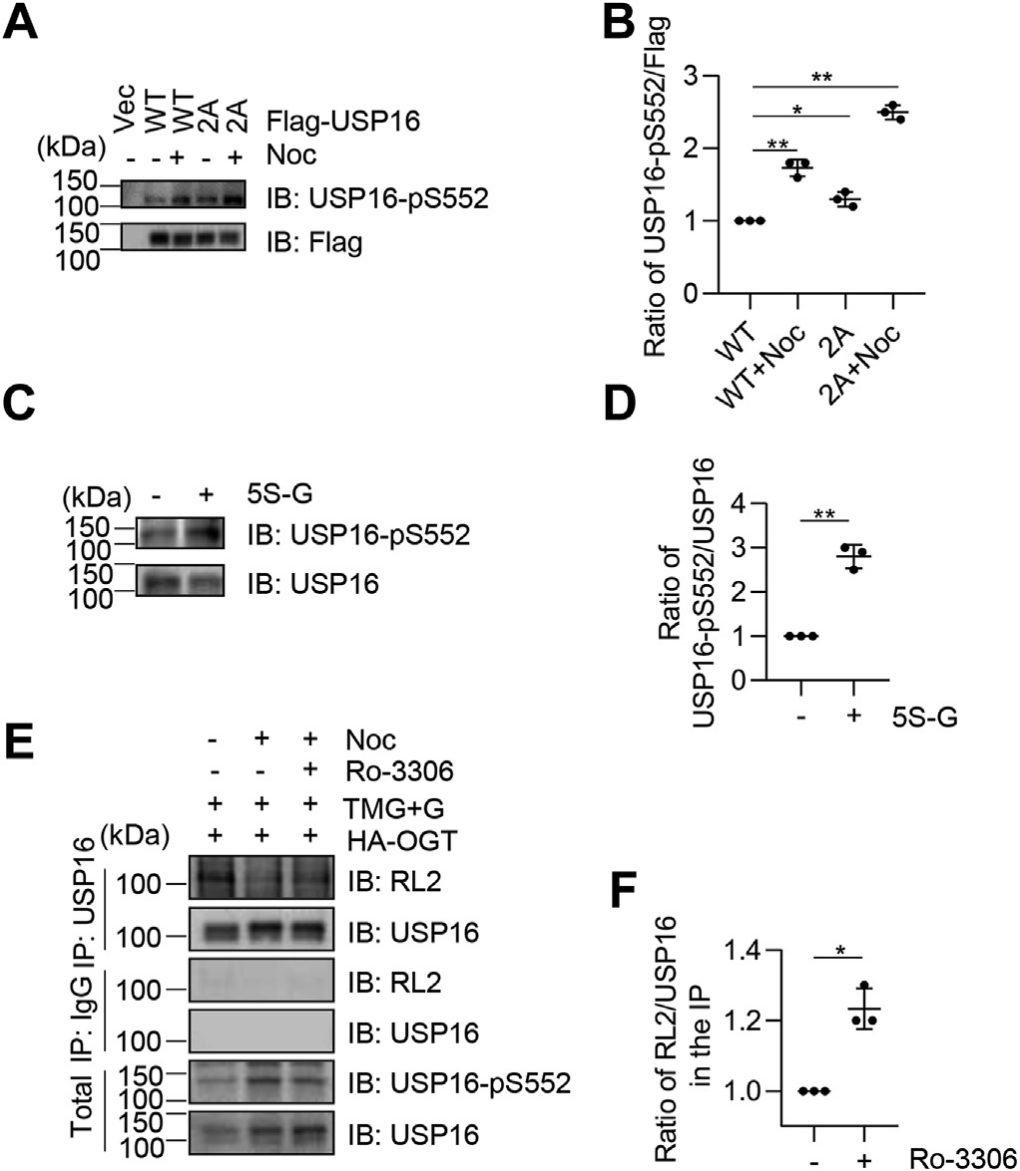
**O-GlcNAc of USP16 antagonizes CDK1-dependent phosphorylation at pSer-552.***A*, HeLa cells were transfected with vectors, Flag-USP16-WT, or -2A, treated or not treated with Noc. The lysates were immunoblotted with the antibodies indicated. *B*, quantitation of the results in (*A*). *C*, cells were treated with 5S-G (OGT inhibitor). *D*, quantitation of the results in (*C*). *E*, cells were synchronized by treating with Noc, then Ro-3306 (CDK1 inhibitor) was added for 2 h. *F*, quantitation of the results in (*E*). A two-way ANOVA was used in (*B*), and the *t* test was used in (*D*) and (*F*). ∗ indicates *p* < 0.05; ∗∗ indicates *p* < 0.01. 5S-G, acetyl-5S-GlcNAc; CDK1, cyclin-dependent kinase 1; GlcNAc, O-linked β-N-acetylglucosamine; OGT, O-GlcNAc transferase; O-Noc, nocodazole; USP16, ubiquitin-specific peptidase 16.

To test this, we transfected Flag-USP16 or the 2A mutant into HeLa cells and isolated USP16 by Flag IP. As shown in Figure 2, *A* and *B*, Ser552 phosphorylation was significantly increased by nocodazole (Noc) treatment, which arrests cells at the M phase. The 2A mutations resulted in increased levels of pSer552 in both asynchronous and mitotic cells (Fig. 2, *A* and *B*), suggesting that O-GlcNAcylation antagonizes USP16 pSer552. This was confirmed by treating cell with the OGT inhibitor 5S-G, which caused an elevated level of pSer552 (Fig. 2, *C* and *D*). The antagonism between USP16 O-GlcNAcylation and phosphorylation was also evident when cells were treated with the CDK inhibitor Ro-3306. Noc treatment caused an increase of pSer552 levels, with a concomitant decrease of USP16 O-GlcNAcylation levels (Fig. 2, *E* and *F*). Ro-3306 treatment reduced pSer552 levels and enhanced USP16 O-GlcNAcylation (Fig. 2, *E* and *F*), suggesting that USP16 O-GlcNAcylation and Ser552 phosphorylation counteract each other.

### USP16 O-GlcNAcylation promotes USP16 nuclear export

**Figure 3:**
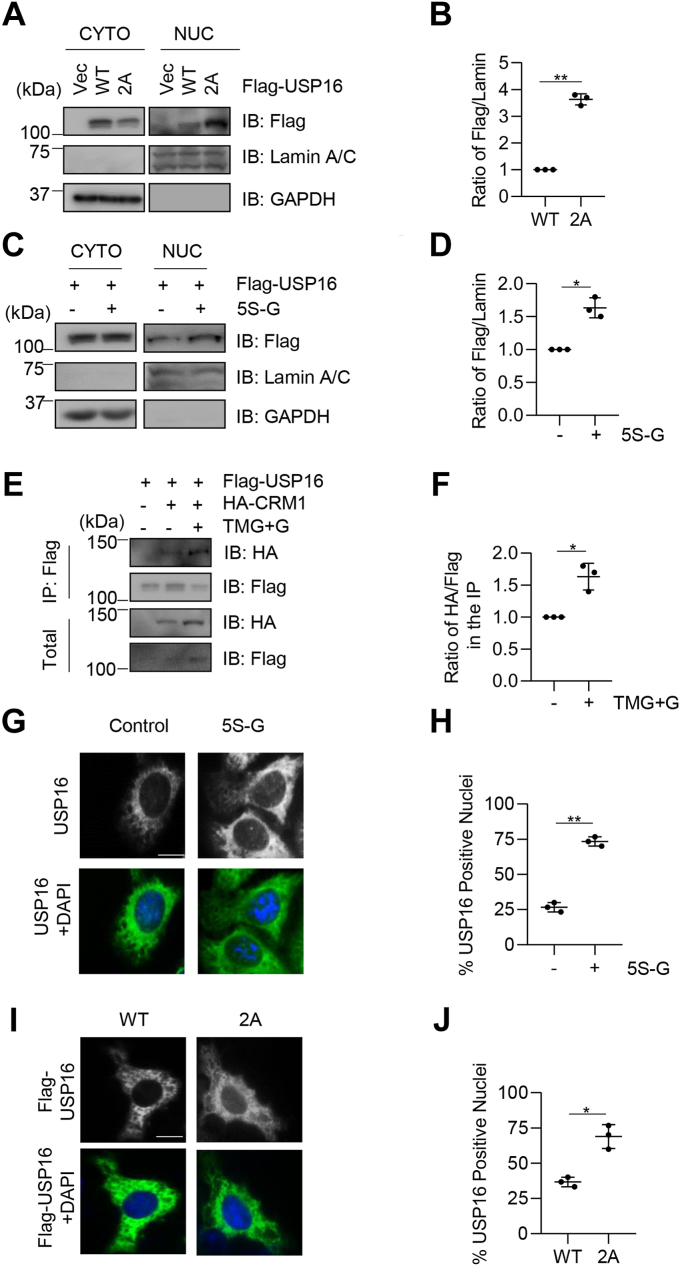
**O-GlcNAc promotes USP16 nuclear export by enhancing USP16-Crm1 affinity.***A*, cells were transfected with vectors, Flag-USP16-WT, or -2A, and the lysates were fractionated into cytosolic and nuclear portions. Immunoblotting was carried out to identify the proteins indicated. *B*, quantitation of the results in (*A*). *C*, cells were transfected with Flag-USP16, treated or not treated with 5S-G (the OGT inhibitor), and then the cytosolic and nuclear fractions were subject to immunoblotting assays. *D*, quantitation of the results in (*C*). *E* and *F*, cells were transfected with Flag-USP16 and HA-Crm1 plasmids, treated with TMG + G. *E*, the cellular lysates were collected and immunoblotted with anti-Flag antibodies. *F*, quantitation of the results in (*E*). *G*, cells were treated with 5S-G or left untreated and stained with anti-USP16 antibodies and DAPI. *H*, quantitation of the results in (*G*). *I*, cells were transfected with Flag-USP16-WT, or -2A, then stained with anti-Flag antibodies and DAPI. *J*, quantitation of the results in (*I*). These experiments were repeated three times, with n = 30. Scale bar: 10 μm. Statistics was carried out with a *t* test. ∗ indicates *p* < 0.05; ∗∗ indicates *p* < 0.01. 5S-G, acetyl-5S-GlcNAc; Crm1, chromosome region maintenance 1; DAPI, 4′,6-diamidino-2-phenylindole; O-linked β-N-acetylglucosamine; TMG, Thiamet-G; USP16, ubiquitin-specific peptidase 16; O-GlcNAc.

Our previous report suggests that pSer552 facilitates USP16 nuclear localization by blocking its interaction with Crm1 (or exportin 1), the protein machinery responsible for protein nuclear export (27). To determine whether USP16 O-GlcNAcylation reverses the effect of pSer552, we measured the subcellular distribution of USP16. We found that the 2A mutation, which abolishes its O-GlcNAcylation, caused a significant increase of USP16 in the nuclear fraction and a significant decrease of USP16 in the cytoplasm (Fig. 3, *A* and *B*). Inhibition of OGT activity by 5S-G also increased the fraction of USP16 in the nucleus (Fig. 3, *C* and *D*). Thus, it appears that USP16 O-GlcNAcylation promotes USP16 nuclear export.

We recently reported that O-GlcNAcylation of YTH domain family 1 enhances YTH domain family 1-Crm1 binding (33). To determine whether the change in the subcellular localization of USP16 is due to impaired interaction with Crm1, we transfected HeLa cells with Flag-USP16 and HA-Crm1. We used TMG + G to increase O-GlcNAcylation, and it strengthened interaction between USP16 and Crm1 (Fig. 3, *E* and *F*). This result suggests that O-GlcNAc decreases nuclear USP16 by promoting USP16-Crm1 binding.

The effect of USP16 nuclear export by O-GlcNAcylation was also observed under microscopy. 5S-G treatment markedly increased the nuclear localization of USP16 (Fig. 3, *G* and *H*), and the USP16-2A mutation increased the nuclear localization of USP16 (Fig. 3, *I* and *J*). Together, our results demonstrate that O-GlcNAcylation promotes USP16 nuclear export, probably by enhancing its interaction with the nuclear exporter Crm1.

### O-GlcNAcylation regulates the DUB activity of USP16

**Figure 4:**
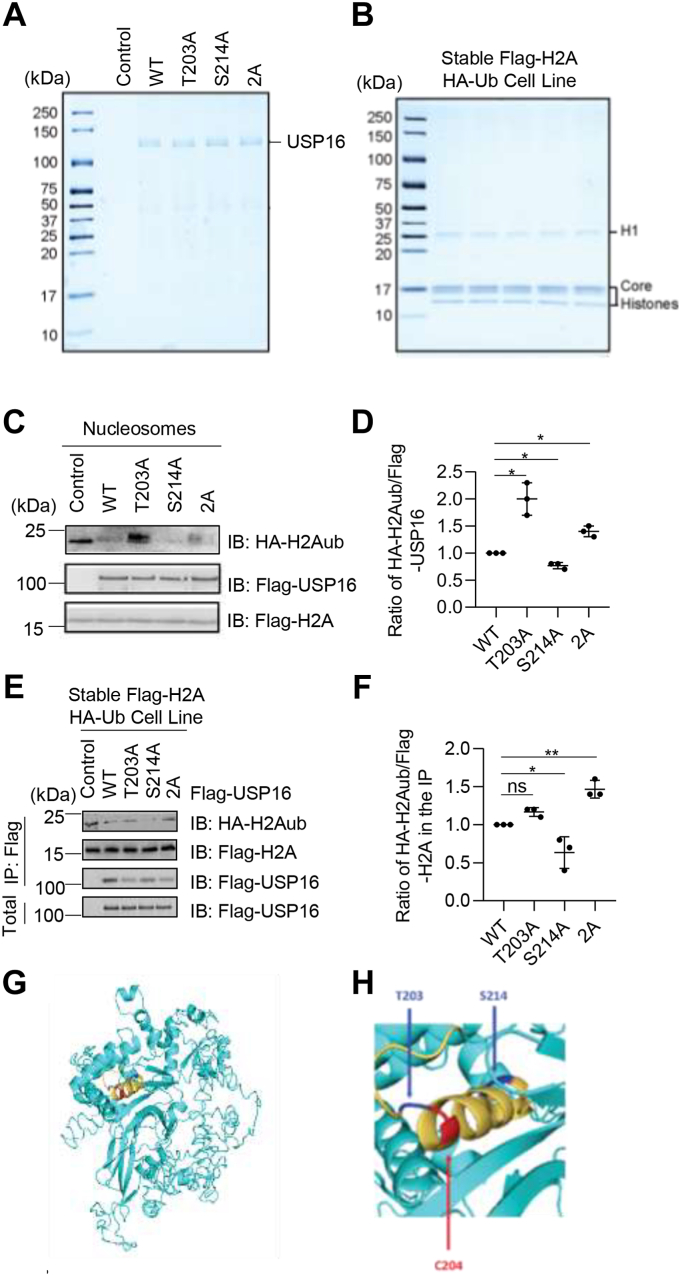
**USP16 O-GlcNAcylation promotes mitotic H2Aub deubiquitination *in vitro* and in cells.***A*, purified USP16 proteins and its mutant forms. *B*, substrates used in the *in vitro* histone deubiquitination assay. *C*, *in vitro* analysis. Recombinant USP16-WT or mutant proteins from (*A*) were incubated with H2Aub-containing nucleosomes from (*B*). And the enzymatic activity was determined with Western blotting analysis. *D*, quantitation of the results in (*C*). *E*, in cell activity analysis. Stable Flag-H2A HA-Ub cell lines were transfected with Flag-USP16-WT and mutants. The cell extracts were immunoprecipitated with anti-FLAG antibodies under denaturing conditions. *F*, quantitation of the results in (*E*). *G*, homology modeling showing the position of USP16 O-GlcNAcylation sites. The alpha helix where the catalytic triad Cys204 is located is shown in *gold*. *H*, detailed position of USP16 O-GlcNAcylation sites Thr203 and Ser214 in relation to the critical catalytic residue Cys204. A one-way ANOVA was used in the statistics. ∗ indicates *p* < 0.05; ∗∗ indicates *p* < 0.01. USP16, ubiquitin-specific peptidase 16.

We are intrigued by the possibility that O-GlcNAcylation may affect the deubiquitination activity of USP16, as the two O-GlcNAcylation sites Thr203 and Ser214 flank Cys204, the amino acid required for USP16 deubiquitination activity. To explore this hypothesis, we purified USP16-WT, T203A, S214A, and 2A proteins that were expressed in 293T cells (Fig. 4*A*). We used mononucleosomes that were purified from a HeLa cell line overexpressing Flag-H2A and HA-ubiquitin as substrates (Fig. 4*B*). *In vitro* deubiquitination assay was performed as detailed in our previous publications (34). Anti-HA immunoblots, which detected the ubiquitin molecule attached to the nucleosomes, revealed that while USP16-WT efficiently deubiquitinates uH2A, the T203A mutation completely abolishes its deubiquitination activity (Fig. 4, *C* and *D*). While S214A mutation has little effect on the deubiquitination activity of USP16, the deubiquitination activity of the 2A mutant is somewhere between T203A and T214A (Fig. 4, *C* and *D*). This result suggests that T203 and S214 have opposite effects on the deubiquitination activity of USP16. Thus, the coordination of O-GlcNAcylation on T203 and S214 may fine-tune the deubiquitination activity of USP16.

The opposite effect of Thr203 and Ser214 O-GlcNAcylation on USP16 was also observed in cells. When a HeLa cell line stably expressing Flag-H2A and HA-Ub was transfected with WT or USP16 with mutations at Thr203 and/or Ser214, the levels of uH2A, which were detected by anti-HA immunoblots, were significantly reduced by expressing WT USP16 (Fig. 4, *E* and *F*). The T203A mutant USP16 was less active while the S214A mutant was more active than WT (Fig. 4, *E* and *F*). The 2A mutant was also less active than WT and the S214A mutant (Fig. 4, *E* and *F*). When using the PyMOL tool to visualize the spatial position of USP16 O-GlcNAc sites, Thr203 was found to localize at the end of the alpha helix (in gold), and Ser214 was found to localize in the last turn of the alpha helix (Fig. 4, *G* and *H*). The catalytic-required amino acid Cys204 was localized in the N-terminal first turn of the alpha helix. Future research is needed to investigate how Thr203 and Ser214 O-GlcNAcylation coordinate to regulate the enzymatic activity of USP16, particularly in response to changes of cellular glucose levels.

### USP16 O-GlcNAcylation promotes H2A and PLK1 deubiquitination in cells

**Figure 5:**
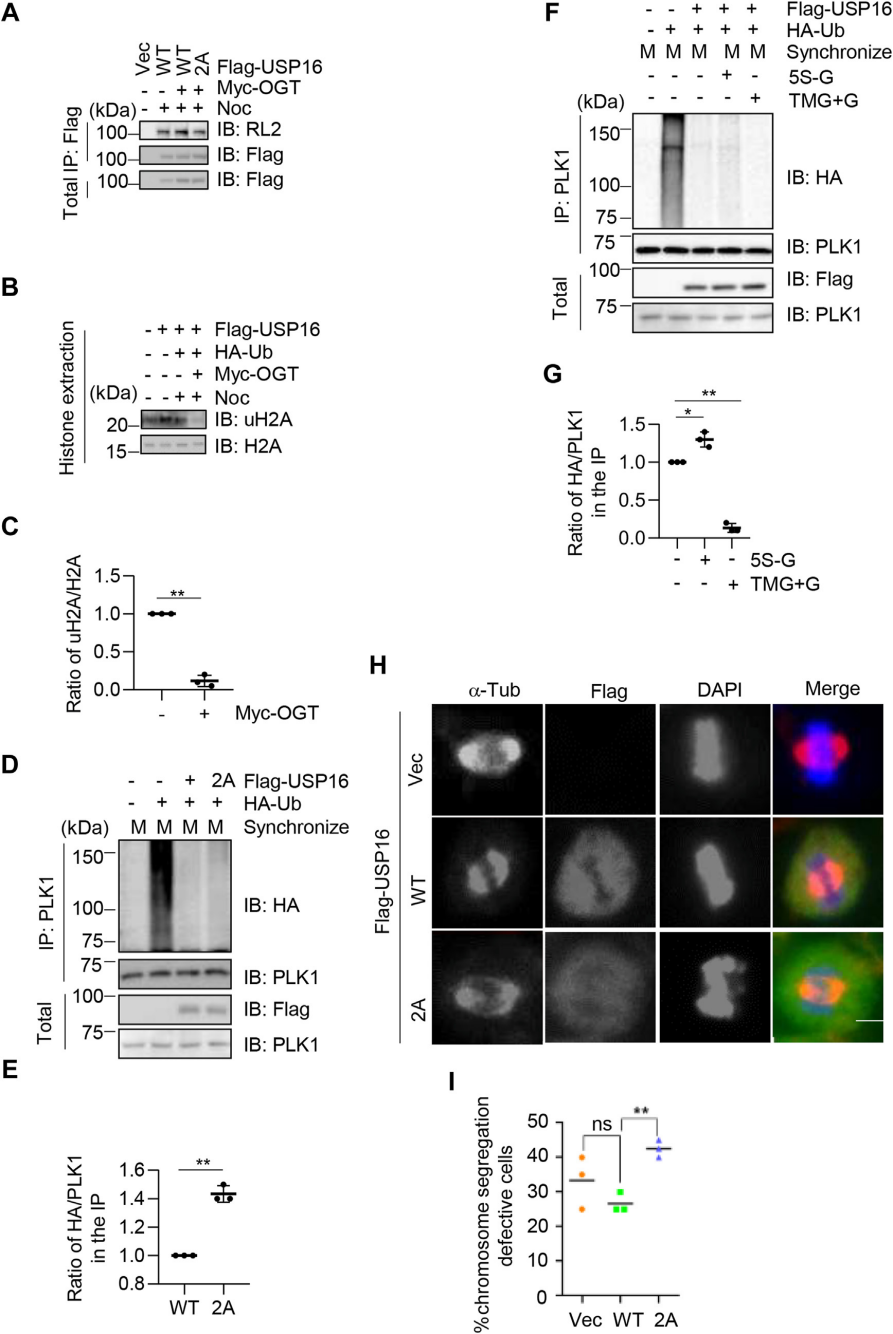
**O-GlcNAc of USP16 promotes PLK1 ubiquitination and regulates mitotic chromosome segregation.***A* and *B*, USP16 O-GlcNAcylation decreases uH2A. Cells were transfected with Flag-USP16 and Myc-OGT plasmids and synchronized with nocodazole (Noc) in (*A*). Immunoprecipitation and immunoblotting show that USP16 O-GlcNAcylation is upregulated upon OGT transfection. In (*B*) cells were transfected with Flag-USP16, HA-Ub, and Myc-OGT plasmids. Histone extraction was carried out to examine uH2A levels after OGT transfection. *C*, quantitation of the results in (*B*). *D*, cells were transfected with Flag-USP16-WT or -2A, together with HA-Ub plasmids. Cells were then mitosis arrested, and the cell lysates were immunoprecipitated with anti-PLK1 antibodies and immunoblotted with the antibodies indicated. *E*, quantitation of the results in (*D*). *F*, mitosis-arrested cells were treated with OGT inhibitor (5S-G), or OGA inhibitor (TMG) together with glucose. *G*, quantitation of the results in (*F*). *H*, cells were transfected with Flag-USP16-WT or -2A, and costained with anti-Flag, anti-α-tubulin antibodies, together with DAPI. *I*, quantitation of the results in (*H*). These experiments were repeated three times, with n = 40 to 50. Scale bar = 10 μm. A *t* test was used in (*C*) and (*E*). A one-way ANOVA was used in (*G*) and (*I*). ∗ indicates *p* < 0.05; ∗∗ indicates *p* < 0.01. 5S-G, acetyl-5S-GlcNAc; DAPI, 4′,6-diamidino-2-phenylindole; ns, nonspecific; O-GlcNAc, O-linked β-N-acetylglucosamine; OGT, O-GlcNAc transferase; PLK1, Polo-like kinase 1; USP16, ubiquitin-specific peptidase 16.

To determine whether O-GlcNAcylation regulates the ubiquitination levels of USP16 substrates, we conducted a series of experiments in HeLa cells. First, we transfected Flag-USP16, HA-Ub, and Myc-OGT into the cells (Fig. 5*A*) and used Noc to synchronize them into the M phase. We found that overexpression of OGT led to an increase in the levels of USP16 O-GlcNAcylation (Fig. 5*A*), which was abolished when the 2A mutant was expressed (Fig. 5*A*). Next, we overexpressed OGT in cells and examined the levels of uH2A. Our results showed a marked decrease in uH2A (Fig. 5, *B* and *C*), consistent with previous results that O-GlcNAcylation stimulates USP16 deubiquitination activity.

We also investigated the effects of USP16 O-GlcNAcylation on PLK1, another substrate of USP16 during mitotic progression (28). To determine whether USP16 O-GlcNAc also affects the ubiquitination level of PLK1, we synchronized the cells at early mitosis, as localization of PLK1 to kinetochore during early mitosis will initiate kinetochore-microtubule attachment for chromosome alignment. Cells were transfected with USP16-WT or 2A mutants, and the levels of PLK1 ubiquitination was then examined (Fig. 5, *D* and *E*). We observed a marked increase in the level of PLK1 ubiquitination in the 2A mutant-transfected cells, consistent with our study that USP16 O-GlcNAcylation promotes its DUB activity. Furthermore, we treated cells with the OGT inhibitor 5S-G and found a marked increase in PLK1 ubiquitination levels (Fig. 5, *F* and *G*), while treating cells with the OGA inhibitor TMG combined with glucose supplement decreased the levels of PLK1 ubiquitination (Fig. 5, *F* and *G*). Thus, O-GlcNAcylation also regulates the ubiquitination level of PLK1 in cells.

Previous studies reveal that USP16 mediates the deubiquitination of PLK1 and the subsequent chromosome alignment (28). To determine whether defects of USP16 O-GlcNAcylation affect chromosome segregation, we monitored mitotic defects in cells transfected with the USP16-2A mutant (Fig. 5, *H* and *I*). Our results showed that cells transfected with WT-USP16 exhibited proper chromosome alignment during segregation, whereas cells transfected with the 2A mutant displayed misaligned chromosomes (Fig. 5, *H* and *I*). These findings suggest that O-GlcNAcylation affects the ubiquitination levels of USP16 substrates and regulates cell cycle progression.

### USP16 O-GlcNAcylation is essential for cytokinesis

**Figure 6:**
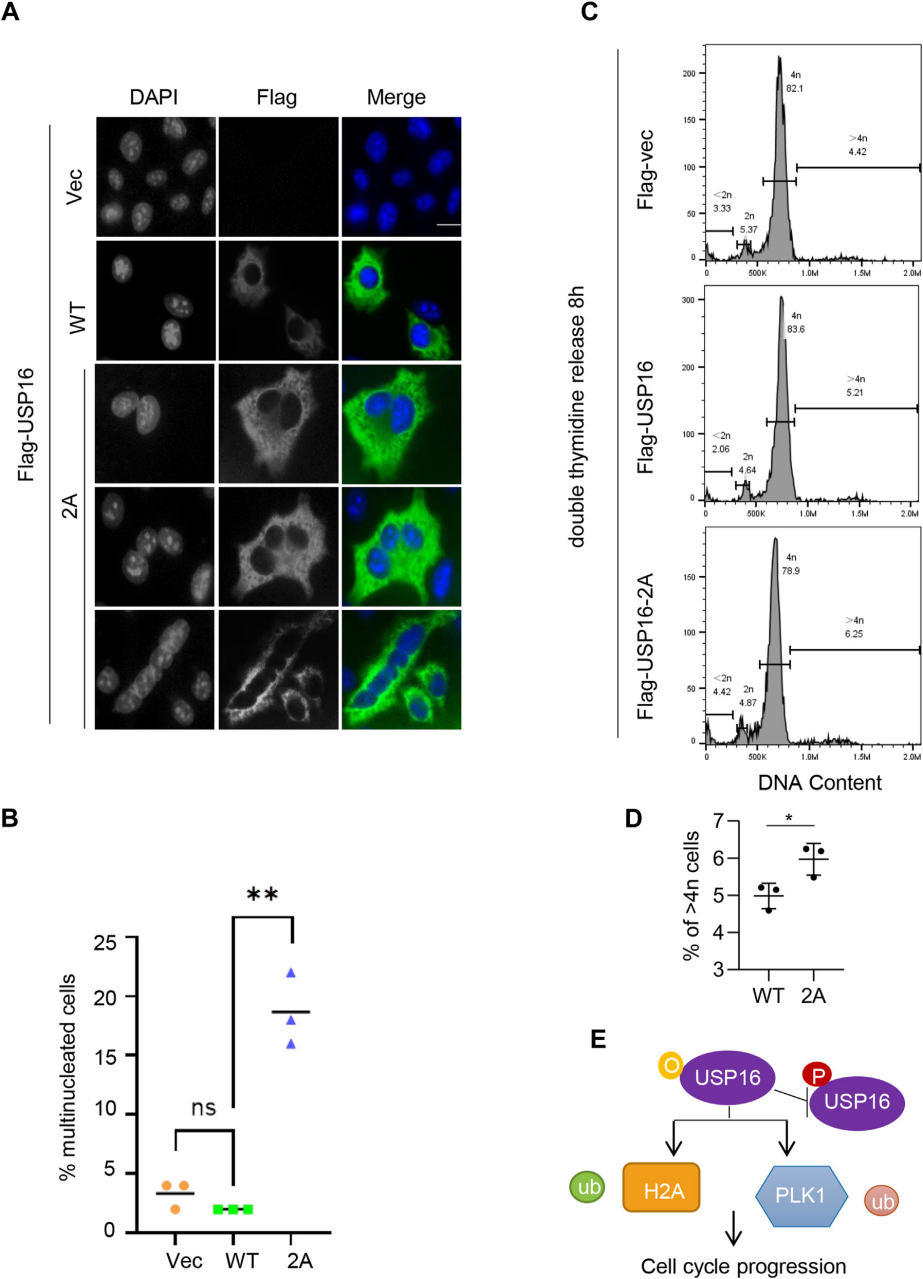
**USP16 O-GlcNAcylation promotes cytokinesis.***A*, cells were transfected with Flag-USP16-WT or USP16-2A and costained with anti-Flag antibodies together with DAPI. *B*, binucleated or multinucleated cells were quantitated in (*B*). These experiments were repeated three times, with n = 40 to 50. A *t* test was used in the statistics. ∗∗*p* < 0.01. Scale bar = 10 μm. *C* and *D*, cells were transfected with Flag-USP16-WT, or -2A, synchronized by double thymidine, and then released for 8 h. The cells were then collected for flow cytometry analysis. *D*, quantitation of (*C*). A one-way ANOVA was used in the statistics. ∗*p* < 0.05. *E*, a model showing the potential role of O-GlcNAcylation in regulating H2Aub and PLK1 to ensure cell cycle progression. DAPI, 4′,6-diamidino-2-phenylindole; ns, nonspecific; PLK1, Polo-like kinase 1.

Cytokinesis is the final stage of mitosis where the cytoplasm is distributed into two daughter cells. Defects in chromosome segregation often lead to cytokinesis failure, resulting in binucleated or multinucleated cells (35). We discovered that cells transfected with the USP16-2A mutant have a significant larger portion of binucleated or multinucleated cells (Fig. 6, *A* and *B*). We further synchronized the cells in cytokinesis as described (36) and used flow cytometry to quantify the cytokinetic cells in USP16-WT and USP16-2A cells (Fig. 6, *C* and *D*). The result shows that the USP16-2A transfected cells increased cytokinetic portion, suggesting that chromosome segregation defects caused by expression of USP16-2A led to cytokinetic failure.

## Discussion

In this paper, we report that O-GlcNAcylation of USP16 promotes its DUB activity toward uH2A and PLK1 and regulates USP16 localization through Crm1 (Fig. 6*E*). A recent investigation revealed that USP16 contains a noncanonical nuclear localization signal at the 437 to 459 amino acid region and a nuclear export signal at the 572 to 581 amino acid region (ISNGFKNLNL) (37). As pS552 is close to the nuclear export signal and found to promote the nuclear localization of USP16 (27), our work further implicates the role of O-GlcNAcylation in regulating USP16 localization and function. Considering USP16 and uH2A play pivotal roles during animal development, as well as in K-RAS-driven lung cancer (23), castration-resistant prostate cancer (24), an Alzheimer’s model (26), transcription-replication conflicts caused by replication stress (38), and fly longevity and healthy lifespan (39), our work may have implications in future therapeutic interventions targeting USP16.

Here we aim to shed light on OGT’s substrates in epigenetics. Previously, we identified three mitotic substrates of OGT: Cdh1, MYPT1, and PLK1. Cdh1 O-GlcNAcylation promotes anaphase-promoting complex/cyclosome activity and thus mitotic exit (31), MYPT1 O-GlcNAcylation promotes PLK1 activity and centrosome separation (32), and PLK1 O-GlcNAcylation ensures correct chromosome segregation (40). In this study, we further identify USP16 as another OGT substrate that regulates uH2A. Notably, in all three cases of Cdh1, MYPT1, and USP16, O-GlcNAcylation counteracts CDK1-mediated phosphorylation. For instance, Cdh1 O-GlcNAcylation at Ser39, Ser40, and Ser42 opposes CDK1-induced phosphorylation, as measured by the MPM-2 antibody (targeting the pS/T P motif, CDK1 substrate consensus site), and Cdh1 Ser40 is both O-GlcNAcylated and phosphorylated (31). Similarly, MYPT1 O-GlcNAcylation at Thr577, Ser585, Ser589, and Ser601 antagonizes pSer473, which are about 100 amino acids apart (32), while USP16 O-GlcNAcylation at Thr203 and Ser214 acts against pSer552, which are almost 300 amino acids away. Perhaps the underlying mechanism is that CDK1-mediated phosphorylation events are on the rise during mitosis, while both OGT protein levels (41) as well as OGT mRNA levels (42) are declining. Although recent chemoproteomic screens identified many new OGT substrates during the cell cycle (43, 44), a unified view of the relationship between O-GlcNAc and CDK1-induced phosphorylation is still lacking. The identification of more substrates in the future may provide a more consistent view.

PLK1 is a pivotal mitotic kinase. It is worth noting how OGT regulates PLK1 in different aspects. In our previous papers, we described that O-GlcNAcylation of MYPT1, the targeting subunit of the protein phosphatase for PLK1, promotes PLK1 activity (32). We also demonstrated that PLK1 itself is a direct target of OGT (40). Here, we further show that O-GlcNAcylation of USP16, the DUB of PLK1, enhances PLK1 deubiquitination, ensuring correct mitotic progression. As PLK1 is central for mitosis, there might be other PLK1-associated factors that are regulated by OGT.

Many histone codes, including H2BS112, H3pS10, and H4S47, have been shown to be regulated by OGT. In this study, we describe the DUB for uH2A is modulated by OGT. Notably, Thr203 and Ser214 exert opposing effects on uH2A, albeit the 2A mutant displays an overall defective DUB activity. The structure of USP16 is yet to be revealed, but homology modeling shows that the two potential O-GlcNAc sites are located on the same helix as the catalytic Cys204 (Fig. 4, *G* and *H*), which impacts its enzymatic function. It is conceivable that the two sites might respond to glucose differently. Alternatively, their effect might correlate with their distance to the catalytic Cys204. In light of the recent finding that USP16 is an ISG15 and a FUBI cross-reactive DUB (45, 46), it will be of interest to examine the effect of USP16 O-GlcNAcylation on ISGylation and deFUBIylation. Further studies are needed to clarify the impact of O-GlcNAc glycosylation on the USP16 protein structure and the underlying mechanisms. Given the complexity of the histone code, which involves numerous readers, writers, and regulators (47), we anticipate that there are additional O-GlcNAcylated epigenetic factors awaiting discovery.

## Experimental procedures

### Cell culture, antibodies, and plasmids

HeLa cells were purchased from ATCC. The cell lines were validated using STR profiling and free from *mycoplasma* contamination for all experiments. Antibodies: anti-β-actin (Sigma, A5441); anti-HA (Bethyl Laboratories, A190-108A); anti-FLAG (Sigma, F1084); anti-Myc (PTM Bio, #PTM-5390); and anti-USP16 and anti-USP16-pS552 antibodies were described before (27); anti-OGT (Abcam, AB96718);RL2 (Abcam AB2739); anti-Histone H2A (CST #2578); anti-uH2A (Abcam AB193203); and anti-PLK1 (Santa Cruz, SC-17783). *USP16* plasmids were described before (27). *USP16-T203AS214A(2A)* plasmids were generated using specific primers (sequences available upon request) following the manufacturer’s instructions (ClonExpress Ultra One Step Cloning Kit, Vazyme C115). OGT plasmids and antibodies have been described (31).

### IP and immunoblotting assays

IP and immunoblotting experiments were performed as described before (48). The nuclear and cytoplasmic fractionation assay was carried out as described (49). Briefly, cells were lysed in the lysis buffer (10 mM Hepes, pH 7.9, 50 mM NaCl, 0.5 M sucrose, 0.1 mM EDTA, and 0.1% Triton X-100) and then centrifuged to pellet the nuclear fraction. The supernatants were further centrifuged to remove any nuclear debris and were collected as the cytosolic fraction.

The following primary antibodies were used for IB: anti-β-actin (1:10,000), anti-HA (1:1000), anti-FLAG M2 (Sigma) (1:1000), anti-Myc (1:1000), anti-USP16 (1:1000), anti-USP16-pS552 (1:1000), and anti-PLK1 (1:1000). Peroxidase-conjugated secondary antibodies were from JacksonImmuno Research. Blotted proteins were visualized using the ECL detection system (Amersham). Signals were detected by a LAS-4000 and quantitatively analyzed by densitometry using the Multi Gauge software (Fujifilm). All western blots were repeated for at least three times.

### Cell culture synchronization

Chemical utilization: Nocodazole (Noc) (Sigma M1404-50MG) at 100 ng/ml for 16 h; Ro 3306 (CDK1 inhibitor) (MCE HY-12529) at 2 μM for the time indicated; TMG (OGA inhibitor) (Sigma SML0244-5MG) at 5 μM for 24 h; TMG+Glu treatment was utilized as before (30); and 5S-G (OGT inhibitor) was used at 100 μM (prepared at 50 mM in DMSO) for 24 h. Cell synchronization in early mitosis (Fig. 6, *A* and *B*) was carried out as described (28). Briefly, cells were incubated with thymidine (Sigma 89270-5G), then nocodazole to be synchronized to prometaphase. Then nocodazole was washed off, and MG132 (the protease inhibitor) (MCE HY-13259) was added to the medium to synchronize the cells to metaphase.

### Click chemistry

Cells were transfected with Flag-USP16, then treated with 200 μmol/l Ac_3_6AzGlcNAc and 5 μmol/l TMG (Sigma-Aldrich) for 24 h, and treated or not treated with nocodazole (Sigma-Aldrich) for 16 h or 5S-G (a present from Dr Chen lab) for 24 h. Collected cells were lysed with 150 mM lysis buffer (150 mM NaCl, 1 M Tris-HCl (pH 7.5), 0.5 M EDTA, 10% NP-40) containing a protease inhibitor cocktail (Roche) for 1 h at 4 °C. Next, cell lysates were cleared using centrifugation (4 °C; 12,000 rpm; 10 min). The supernatant was reacted with 50 μmol/l DBCO-PEG4-Biotin from Duyouyou Biotechnology, 8 mmol/l urea, 10 mmol/l Hepes (pH 7.9), and Halt protease & phosphatase inhibitor cocktail (100×) from Thermo Fisher Scientific, then the pull-down complex isolated by streptavidin-coupled beads was subjected to Western blotting analysis.

### Indirect immunofluorescence

Indirect immunofluorescence staining was performed as described before (50). Dilutions of primary antibodies were 1:1000 for mouse anti-α-tubulin. Cell nuclei were stained with DAPI.

### Nucleosome preparation and histone deubiquitination assay

Preparation of uH2A-containing nucleosomes was performed as described previously (51). Briefly, Flag-H2A and HA-ubiquitin were transfected into cells to generate a stable cell line named HF29. uH2A-containing nucleosomes were purified from HF29 cells. USP16-WT and mutant plasmids were transfected into 293T cells. USP16-WT and mutant proteins were purified with anti-Flag M2 agarose beads (Sigma, A2220).

Histone deubiquitination reactions were performed as described previously (18). Equal amounts of mononucleosomes were incubated with equal amount of purified USP16 wild-type or mutant proteins in the deubiquitination assay buffer (100 mM Tris-HCl, pH 8.0, 1 mM EDTA, 0.1 mM PMSF, 1 mM DTT, 1 μg/ml aprotinin, 1 μg/ml leupeptin, and 1 μg/ml pepstatin A) at 37 °C for 30 min. The reaction was stopped by adding SDS-PAGE loading buffers and heating. Ubiquitination levels were determined by Western blotting with anti-Flag and anti-HA antibodies.

*In vivo* histone deubiquitination assays were carried out as described under denaturing conditions (18, 34). Stable USP16-knockdown cells expressing *USP16* shRNA were transfected with Flag-USP16-WT and mutant plasmids. Transfected cells were dissolved into denaturing buffers (20 mM Tris, pH 7.4, 50 mM NaCl, 0.5% Nonidet P-40, 0.5% deoxycholate, 0.5% SDS, 1 mM EDTA, and protease inhibitors) by sonication. After centrifugation, ubiquitinated H2A was detected by Western blotting.

### Flow cytometry

HeLa cells were transfected with Flag-vec, Flag-USP16-WT, and Flag-USP16-2A plasmids. For synchronization to the cytokinesis stage, cells were incubated in complete media containing 2 mM thymidine for 18 h. Cells were then returned to normal media for 9 h and then placed back into thymidine media for 17 h. Cells were then released into complete media and harvested at 8 h, as described before (36). Cells were harvested with 0.25% trypsin, fixed in 70% ice-ethanol overnight, and permeabilized by 0.25% Triton X-100 on ice for 15 min. Cells were incubated in 50 μg/ml propidium iodide and 100 μg/ml Rnase A at room temperature for 30 min, followed by DNA content analysis using BD Accuri C6. The DNA content was analyzed using FlowJo software (https://www.flowjo.com/).

### Statistical rational

Experiments were repeated for at least three times. Statistics was carried out using Prism 8 (Graphpad Software, https://www.graphpad.com), with the methods and *p*-values indicated in the figure legends. T-tests were used for two unpaired groups. One-way ANOVA was used when one factor affects the outcome. Two-way ANOVA was used when two factors affect the results.

## Data availability

All data are contained within the manuscript.

## Conflict of interest

The authors declare that they have no conflicts of interest with the contents of this article.
